# Risk of ovarian cancer in relation to use of phenolphthalein-containing laxatives

**DOI:** 10.1054/bjoc.2000.1250

**Published:** 2000-07-03

**Authors:** G S Cooper, M P Longnecker, D P Sandler, R B Ness

**Affiliations:** Epidemiology Branch, National Institute of Environmental Health Sciences, Durham, NC, 27709, USA; University of Pittsburgh Graduate School of Public Health, 130 DeSoto St, 517 Parran, Pittsburgh, PA, 15261, USA

**Keywords:** ovarian cancer, phenolphthalein, laxatives

## Abstract

We examined ovarian cancer risk in relation to use of phenolphthalein-containing laxatives in 410 epithelial ovarian cancer cases and 713 controls. Compared to women who never used a laxative, ever use of a phenolphthalein-containing laxative was not associated with an increased risk of ovarian cancer (odds ratio, OR, 1.1, 95% confidence interval, CI, 0.9–1.4). Risk was slightly, but not significantly, higher with more frequent use (OR 1.2 for 75 or more days of use). When women who used non-phenolphthalein containing laxatives was used as the reference group, the associations were slightly, but not significantly larger (OR 1.4 for any use of phenolphthalein-containing laxatives and OR 1.5 for 75 or more days of use) © 2000 Cancer Research Campaign


*British Journal of Cancer* (2000) **83**(3), 404–406
© 2000 Cancer Research Campaign
doi: 10.1054/ bjoc.2000.1250, available online at http://www.idealibrary.com on IDEAL®

### Risk of ovarian cancer in relation to use of phenolphthalein-containing laxatives

**GS Cooper[1], MP Longnecker[1], DP Sandler[1], RB Ness[2]**

[1]Epidemiology Branch, National Institute of Environmental Health Sciences, Durham NC 27709, USA; [2]University of Pittsburgh Graduate School of Public Health, 130 DeSoto St, 517 Parran, Pittsburgh PA 15261, USA

**Summary** We examined ovarian cancer risk in relation to use of phenolphthalein-containing laxatives in 410 epithelial ovarian cancer cases and 713 controls. Compared to women who never used a laxative, ever use of a phenolphthalein-containing laxative was not associated with an increased risk of ovarian cancer (odds ratio, OR, 1.1, 95% confidence interval, CI, 0.9–1.4). Risk was slightly, but not significantly, higher with more frequent use (OR 1.2 for 75 or more days of use). When women who used non-phenolphthalein containing laxatives was used as the reference group, the associations were slightly, but not significantly larger (OR 1.4 for any use of phenolphthalein-containing laxatives and OR 1.5 for 75 or more days of use) © 2000 Cancer Research Campaign

**Keywords**: ovarian cancer; phenolphthalein; laxatives

Phenolphthalein, the active ingredient in many laxatives, was recently found to be a carcinogen in rats and mice (Dunnick and Hailey 1996; National Toxicology Program, 1996). In the female mice, one of the affected sites was the ovary, and the ovarian lesions (stromal cell hyperplasia and stromal cell tumour) were seen at the lowest dose of phenolphthalein used (3000 ppm in feed, administered continuously for 2 years). This dose is similar to the dose in humans consuming two or more phenolphthalein-containing laxative pills per day. Based on the animal studies, the United States Food and Drug Association reclassified phenolphthalein in over-the-counter medications as 'not generally recognized as safe' (Food and Drug Administration, 1999). Phenolphthalein was voluntarily removed from laxatives sold in the United States by 1998 and similar actions were taken in Japan, Canada, and many European countries (e.g., France, Italy, Germany) (WHO Pharmaceuticals Newsletter, 1998). However, phenolphthalein is contained in a small number of medications licensed in the United Kingdom (personal communication, Medicines Control Agency Information Centre, December 9, 1999) and in other countries (De Smet and Wagenaar, 1998). We report data from a recent population-based case-control study of ovarian cancer conducted in the United States that included detailed information about type and duration of laxative use.

### MATERIALS AND METHODS

From 1994 to 1998, 1253 women aged 20–69 with epithelial ovarian cancer were identified from 39 hospitals in contiguous counties in eastern Pennsylvania, southern New Jersey and Delaware (Ness, in press). Cases were excluded who were diagnosed more than six months prior to interview ($n$ = 296), critically ill or dead ($n$ = 69), or untraceable ($n$ = 15), having 873 women eligible for study. Fourteen physicians did not consent to their patients' participation and 92 women refused to participate, resulting in 767 (88%) completed case interviews of whom 616 had invasive epithelial ovarian cancers and 151 borderline epithelial ovarian tumours. Central pathologic review was conducted on a random sample of 120 cases resulting in agreement for invasiveness in 95% of cases and for cell type in 82% of cases. The original pathologic diagnosis was then used for all cases.

1637 controls younger than 65 were ascertained by random digit dialing and were frequency-matched by 5-year age groups and three-digit telephone exchange to cases. Of these 1215 (74%) were interviewed and 422 declined. 263 controls aged 65–69 were ascertained through Health Care Financing Administration (HCFA) lists and were frequency-matched by age group and zip (postal) code. 152 of these (58%) were interviewed and 111 declined. Details of the sampling and recruitment process have been described (Ness, in press). Therefore, of 1900 eligible potential controls, 1367 (72%) were included. All study subjects gave informed consent for participation.

Standardized 1.5 hour-long home interviews were conducted. A life calendar, marked by important events that participants recalled was used to enhance distant memory. Exposure information was obtained up to the reference date of six months prior to interview. A section of the interview on laxative use was added midway through the study. Approximately half (52.8%, 410 cases and 716 controls) of the participants completed the interview with the laxative section, and there was little difference in the percent of cases (53.5%) and controls (52.4%) who provided these data. Two controls were missing data on telephone area code and so were excluded from the conditional logistic regression models that included this matching variable.

Participants were asked if they had 'ever taken a laxative to help you move your bowels' and if they had taken more then six laxative pills in total up to the reference date. A list of 21 laxatives was provided to elicit more detailed information. Participants were asked the name of every laxative they had used, age at first use and

*Received 9 February 2000*
*Accepted 14 February 2000*

*Correspondence to:* GS Cooper when last used for each, number of days and on average, approximate number used each day. The maximum number of different laxatives used was six. Laxative-specific information was used to determine if a phenolphthalein-containing laxative had ever been used. For such laxatives, we calculated total frequency of use (days), average number of pills per day, cumulative dose and age at first and last use.

We used the National Toxicology Program report to determine phenolphthalein-containing brands of laxatives (National Toxicology Program, 1996), and obtained dose information from the Physicians' Desk Reference for Nonprescription Drugs (Physicians' Desk Reference, 1990). Phenolphthalein-containing laxatives ever used were: Agoral, Alophen, Colax, Correctol, Dialose, Doxidan, Espotabs, Evac-U-Gen, Ex-Lax, Feen-a-Mint, Kondrumel, LaxCaps, Modane and Phenolax.

We used conditional logistic regression to calculate odds ratios (OR) and 95% confidence intervals (CI) to examine association between laxative use and ovarian cancer adjusting for reference age, race (white, non-white), oral contraceptive use (ever, never), tubal ligation, nulligravidity, and telephone area code. The resulting estimates were somewhat weaker than those from unconditional logistic regression. Only the conditional results are presented. Additional adjustment for postal zip code for those age 65 and older had little effect on the estimates. Measures of frequency of use (total days, average number of pills per day, and cumulative dose), and age at first and last use were also examined. The referent group was 'no reported laxative use' in these models, and women who had reported using only a non-phenolphthalein-containing laxative were also included as a separate group. Additional analyses used the women who had used only non-phenolphthalein-containing laxatives as the referent group. This is a much smaller comparison group, but addresses possible confounding by symptoms or indication for use.

### RESULTS

Age was similarly distributed for cases and controls. The median age was 52.5 years among cases and 51 years among controls. Overall, 4% of subjects were aged <30, 13% were 30–39, 29% were 40–49, 30% were 50–59 and 24% were 60–69. Approximately one-third (31.7%) of the study participants reported any laxative use (six or more laxative pills ever before their reference date) and of those, 73.7% had used one or more phenolphthalein-containing laxative. Use of a phenolphthalein-containing laxative was weakly associated (OR 1.1) with an increased risk of ovarian cancer (Table 1). Risk was slightly increased among women who reported 75 or more days of use (OR 1.2), among women who reported an average of two or more pills per day (OR 1.2), and among women in the highest quartile of cumulative dose (adjusted OR 1.2). None of these associations were statistically significant. When the group who used non-phenolphthalein containing laxatives is used as the reference group, these odds ratios are slightly larger: for any use of phenolphthalein-containing laxatives the OR was 1.4 (95% CI 0.9–2.1), for 75 or more days of use the OR was 1.5 (95%, CI 0.9–2.5), for two or more pills per day the OR was 1.5 (95%, CI 0.9–2.4), and for cumulative dose > 10 000 mg the OR was 1.4 (95%, CI 0.9–2.4) Later age at first use was associated with increased ovarian cancer risk, but there was no clear pattern with age at last use.

### DISCUSSION

We observed little association between use of phenolphthalein-laxatives and ovarian cancer risk, although the data did suggest risk was increased with increasing exposure (as measured by number of days used, average number of pills per day, and cumulative dose).

The ovarian lesions seen in the experimental study of phenolphthalein in $B6C3F_1$ mice occurred even at the lowest dose used (3000 ppm in the feed, administered continuously for two years, equivalent to 311 mg $m^{-2}$ body surface area) (Dunnick and Hailey 1996). Use of phenolphthalein-containing laxatives at the prescribed dosage (60–130 mg per pill, 1–2 pills per day) is comparable to dosage in the mouse study (Dunnick and Hailey 1996; National Toxicology Program, 1996).

Phenolphthalein was shown to induce thymic lymphomas in heterozygous p53-deficient mice after only 4 months of exposure, and loss of the p53 wild-type allele was demonstrated in all of the lymphomas (Dunnick et al, 1997). However, ovarian cancer was not seen in this study (Dunnick et al, 1997), and there was no evidence of p53 protein accumulation in the ovarian tumours in the previous study using $B6C3F_1$ mice (Dunnick and Hailey 1996; National Toxicology Program, 1996). Phenolphthalein has oestrogenic properties, and another potential mechanism through which phenolphthalein could effect ovarian cancer is through its interaction with the estrogen-receptor (Ravdin et al 1987).

A limitation of this study is the retrospectively collected information about laxative use. Also, the number of very high dose exposures, as has been reported in cases of bulimia and other situations of laxative abuse (Bo-Linn et al, 1983; Bytzer et al, 1989) was very small (only two women reported use of 10 or more pills per day), so we had limited power to detect effects in this group. We did not observe any increased risk of ovarian cancer among users of laxatives that did not contain phenolphthalein, indicating that our results were unlikely to have been affected by confounding by indication for use or symptoms, or by a differential recall bias (over-reporting of laxative use by cases or under-reporting by controls). A link between laxative use and ovarian cancer has not been widely discussed in the media, decreasing the likelihood that differential recall occurred in this study. The data collection included detailed information on specific brands and amount of use. Another strength is that it is a large, population-based study.

Most western European countries now prohibit sale of phenolphthalein-containing laxatives. A limited number of medications available in the United Kingdom contain phenolphthalein. If phenolphthalein continues to be used in some countries, additional epidemiologic studies should be conducted to examine its contribution to ovarian cancer risk. The studies would need to be very large to have adequate power to detect the relatively weak associations observed in our study. This is a commonly used over-the-counter medication for which alternatives are available, however, so it is a potentially important modifiable exposure. More than 20% of study subjects reported using a phenolphthalein-containing laxative. Use for 180 or more days, or a cumulative dose of ≥20 000 mg was reported by 20% of the women who used phenolphthalein-containing laxatives and by 5% of all study subjects. The feasibility and necessity of additional epidemiologic studies would be reduced if pharmaceutical manufactures voluntarily withdraw phenolphthalein-containing laxatives throughout the world.

*© 2000 Cancer Research Campaign*

**Table 1** Phenolphthalein-containing laxative use in relation to ovarian cancer risk

| Category | Cases *n*[a] | Cases % | Controls *n*[a] | Controls % | Adjusted OR[b] | 95% CI[b] |
|---|---|---|---|---|---|---|
| Laxative use[c] | | | | | | |
| None | 282 | 68.8 | 487 | 68.2 | 1.0 | referent |
| Non-phenolphthalein | 27 | 6.6 | 66 | 9.2 | 0.8 | 0.5–1.2 |
| Phenolphthalein | 101 | 24.6 | 161 | 22.6 | 1.1 | 0.9–1.4 |
| Phenolphthalein-laxatives | | | | | | |
| Total days used | | | | | | |
| 1–9 | 21 | 21.0 | 36 | 22.5 | 1.1 | 0.7–1.7 |
| 10–24 | 21 | 21.0 | 42 | 26.3 | 1.0 | 0.6–1.6 |
| 25–74 | 24 | 24.0 | 37 | 23.1 | 1.1 | 0.7–1.7 |
| ≥ 75 | 34 | 34.0 | 45 | 28.1 | 1.2 | 0.8–1.7 |
| (trend *P* value) | | | | | | (0.36) |
| Average number pills per day | | | | | | |
| < 2 | 64 | 67.4 | 103 | 66.9 | 1.1 | 0.8–1.4 |
| ≥ 2 | 31 | 32.6 | 51 | 33.1 | 1.2 | 0.8–1.8 |
| Cumulative dose (mg)[d] | | | | | | |
| < 1000 | 19 | 20.0 | 43 | 27.9 | 0.9 | 0.6–1.5 |
| 1000–2999 | 23 | 24.2 | 36 | 23.4 | 1.1 | 0.7–1.7 |
| 3000–10 000 | 25 | 26.3 | 37 | 24.0 | 1.1 | 0.8–1.7 |
| > 10 000 | 28 | 29.5 | 38 | 24.7 | 1.2 | 0.8–1.7 |
| (trend *P* value) | | | | | | (0.30) |
| Age first use (years) | | | | | | |
| ≤ 15 | 26 | 26.3 | 48 | 30.4 | 0.9 | 0.6–1.4 |
| 16–24 | 25 | 25.3 | 58 | 36.7 | 0.9 | 0.6–1.4 |
| 25–34 | 25 | 25.3 | 30 | 19.0 | 1.3 | 0.9–1.9 |
| ≥ 35 | 23 | 23.2 | 22 | 13.9 | 1.5 | 0.9–2.2 |
| (trend *P* value) | | | | | | (0.10) |
| Age last use (years) | | | | | | |
| ≤ 39 | 31 | 31.3 | 60 | 38.0 | 1.0 | 0.7–1.4 |
| 40–54 | 38 | 38.4 | 58 | 36.7 | 1.2 | 0.8–1.7 |
| ≥ 55 | 30 | 30.3 | 40 | 25.3 | 1.1 | 0.8–1.7 |
| (trend *P*-value) | | | | | | (0.35) |

[a]Number (cases, controls) with missing data: total days used (1,1); number of pills per day (6,7); cumulative dose (6,7); age first use (2,3); age last use (2,3). [b]OR, Odds ratio; CI, Confidence interval. Conditional logistic regression model, adjusting for age, race, oral contraceptive use, tubal ligation, nulligravidity, and telephone area code. One control with missing data on tubal ligation excluded. No laxative use is the referent group; use of non-phenolphthalein containing laxative is also included in the models with frequency (days, pills or dose) of use, age first use, or age last use of phenolphthalein-containing laxatives. [c]Use of six or more laxative pills before reference age. [d]Summation across all phenolphthalein-containing laxatives of mg phenolphthalein per pill times average number of pills per day times total number of days used

### REFERENCES

Bo-Linn GW, Santa Ana CA, Morawski SG and Fordtran JS (1983) Purging and caloric absorption in bulimic patients and normal women. *Ann Intern Med* **99**: 14–17

Bytzer P, Stokholm M, Andersen I, Klitgaard NA and Schaffalitzky de Muckadell OB (1989) Prevalence of surreptitious laxative abuse in patients with diarrhoea of uncertain origin: a cost benefit analysis of a screening procedure. *Gut* **30**: 1379–1384

De Smet PH and Wagenaar HW (1998) Weight reduction preparations from Thailand: unexpected complication for the traveler. *Ned Tijdschr Geneeskd* **142**: 2798–2800

Dunnick JK and Hailey JR (1996) Phenolphthalein exposure causes multiple carcinogenic effects in experimental model systems. *Cancer Res* **56**: 4922–4926

Dunnick JK, Hardisty JF, Herbert RA, Seely JC, Furedi-Machacek EM, Foley JF, Lacks GD, Stasiewicz S and French JE (1997) Phenolphthalein induces thymic lymphomas accompanied by loss of the p53 wild type allele in heterozygous p53-deficient (±) mice. *Toxic Pathol* **25**: 533–540

Food and Drug Administration. Laxative drug products for over-the-counter human use (1999). *Federal Register* **64**: 4535–4540

National Toxicology Program (1996) NTP technical report on the toxicology and carcinogenesis studies of phenolphthalein (CAS no. 77–09–8) in F344/N rats and B6C3F1R mice (feed studies). Research Triangle Park, NC: US Dept of Health and Human Services, Public Health Service, National Institutes of Health; Springfield, VA.: Available from the National Technical Information Service

Ness RB, Grisso JA, Klapper J, Schlesselman JJ, Silberzweig S, Vergona R, Morgan M, Wheeler JE and the SHARE Study Group (in press). Risk of ovarian cancer in relation to estrogen and progestin dose and use characteristics of oral contraceptives. *Am J Epidemiol*

Physicians' Desk Reference for Nonprescription Drugs and Dietary Supplements. 11th edition. Montvale, NJ: Medical Economics Company, Inc. 1990

Ravdin PM, van Beurden M and Jordan VC (1987). Estrogenic effects of phenolphthalein on human breast cancer cells in vitro. *Breast Cancer Res Treat* **9**: 151–154

WHO Pharmaceuticals Newsletter (1998). Laxatives containing phenolphthalein – voluntary withdrawal. Nos. 1–2, 4–15

 *© 2000 Cancer Research Campaign*
